# Phytotoxic Effects of Antibiotics on Terrestrial Crop Plants and Wild Plants: A Systematic Review

**DOI:** 10.1007/s00244-021-00893-5

**Published:** 2021-10-20

**Authors:** Matilde Carballo, Antonio Rodríguez, Ana de la Torre

**Affiliations:** Animal Health Research Centre, National Institute for Agricultural and Food Research and Technology (INIA), Valdeolmos, Madrid, Spain

## Abstract

This review examines the state of knowledge on the phytotoxic effects of antibiotics on terrestrial crop plants and wild (non-crop) plants with the goal of evaluating differences in their sensitivity. This is important because environmental risk assessments of antibiotics currently consider their potential effects only on crop species but not wild species. Overall, we analysed 275 datasets consisting of antibiotic-plant species-endpoint combinations for germination (mg/L) and 169 datasets for plant growth (elongation and biomass) (mg/kg). EC10 and EC50 of each parameter were compared using a quotient approach, in which the geometric mean and the 5th percentile of the crop data were divided by wild data. Quotients were > 1 for elongation growth, suggesting that wild species were more sensitive than crops, while they were < 1 for biomass growth, suggesting quite the contrary. However, < 1% of the data in each dataset came from wild species, preventing definitive conclusions. Merging crop and wild data to evaluate differences in sensitivity among classes of antibiotics and plant families, we found using a linear mixed effect model and post hoc test that plants were most sensitive to phenicol and least sensitive to macrolides and tetracyclines. Further work must be conducted to gain a better understanding of the phytotoxic effects of antibiotics on terrestrial wild plants and subsequently assess whether the current approach to environmental risk assessment of antibiotics is sufficient to protect plant biodiversity.

Antibiotics play a major role in the maintenance of public and animal health. However, excessive use of antibiotics has resulted in the accumulation of micro-contaminants in soil and water ecosystems over the last 20 years (Grenni et al. 2018). In 2017, the European Union (EU) reported the use of nearly 5930 types of antibiotics in livestock farming (EMA 2019). These antibiotics are poorly absorbed in the gut of livestock animals and as much as 90% of certain antibiotics may be excreted in manure, resulting in the accumulation of micro-contaminants in agro-ecosystems (Kumar et al. 2005). In the last decade, antibiotics have been found in soil matrices of areas fertilised with manure and areas used for grazing (0.4 ng to 25 mg per kg of soil) (Cycon et al. 2019).

The accumulation of antibiotics in the soil can alter the structure and activity of microbial communities, increase the abundance of resistance genes in soils (Llor and Bjerrum, 2014; Carvalho et al., 2014), as well as inhibit the growth and performance of plants (Cycon and Piotrowska-Seget 2019; Kumar et al. 2012). Antibiotics can exert phytotoxic effects directly, for example, by decreasing the rate of respiration or synthesis of chlorophyll, as well as indirectly by disbalancing plant–microbe symbiotic relationships (Kumar et al. 2005; Dolliver et al., 2008; Grote et al. 2007; Kuchta et al. 2009; Carter et al. 2014; Carvalho et al. 2014).

Phytotoxicity of antibiotics can be observed in crop species, as well as in wild (non-crop) species present in pastureland and adjacent habitats such as field margins. Such non-crop species are important since they provide a wide range of ecosystem services to agro-ecosystems, including provisioning, regulating, and supporting services (Arts et al. 2015; Boutin et al. 2014). These diverse floral resources (e.g. forbs, legumes) can support pollinator services by providing habitats to bumblebees, hoverflies, butterflies, and honey bees that pollinate native plants in grasslands, thereby enhancing the pollination of agricultural crops (Hendrickson and Sanderson 2017; Kaluza et al. 2017). Therefore, the protection of wild (non-crop) species is critical to preserving biodiversity, as stated in the recommendations of the European Green Deal, which aims to protect, conserve, and enhance the natural capital of the EU (European Commission 2019).

In 2010, the European Food Safety Authority published a framework to identify specific protection goals for the biological community based on ecosystem services that can be affected by plant protection products (PPP), such as conserving biodiversity (European Food Safety Authority 2010, 2014; European Food Safety Authority 2016). Furthermore, stakeholders at a workshop of the Society of Environmental Toxicology and Chemistry (SETAC) in Europe concurred that wild (non-crop) species need to be protected at the level of the population or higher, and that species abundance, biomass, and cover are important attributes associated with maintenance of ecosystem services (Arts et al. 2015). During that workshop, several important knowledge gaps were identified to address these goals. For example, only crop species, not wild plants, are typically used in laboratory and greenhouse experiments to test phytotoxicity of plant protection products (PPPs), and these tests form the basis of legally mandated environmental risk assessments (ERAs). Stakeholders have expressed concern about the lack of information about wild species, leading to doubts about whether ERAs can protect biodiversity. A literature review of studies on the phytotoxicity of PPPs towards crop and wild plant species (Christl et al. 2019) found no consistent sensitivity differences between crop and wild plant species, implying that ERAs of PPPs can adequately protect plant species biodiversity.

Although there is sufficient information on phytotoxicity caused by plant protection products, very few studies have focussed on the potential phytotoxic effects of antibiotics on plants. In this study, we conducted a systematic review of the information available on the phytotoxic effects of antibiotics on terrestrial crop and wild plant species. Our goal was to gain a better understanding of the sensitivity of crop and wild plant species to antibiotics and thereby assess whether ERAs of veterinary medicines are robust enough to protect plant species biodiversity.

## Methods

For this descriptive, cross-sectional study, we systematically examined electronic databases, including PubMed, Scopus, Web of Science, and Google Scholar, to identify studies published between January 1980 and March 2020 that examined the phytotoxic effects of antibiotics used in veterinary medicine on terrestrial plants. Data retrieval was conducted using the direct query and access method.

This systematic review was performed based on the guidelines recommended in the Joanna Briggs Institute Reviewer’s Manual (2017). The aim of this review was to gain a better understanding of the sensitivity of crop and wild plant species by assessing the number of papers published on this subject, the most commonly studied antibiotics and plant species, the different assay methods used to quantify toxicity, the endpoints of those assays, the time periods during which the toxic effects were measured, and the type of samples used for analysis.

**Fig. 1:**
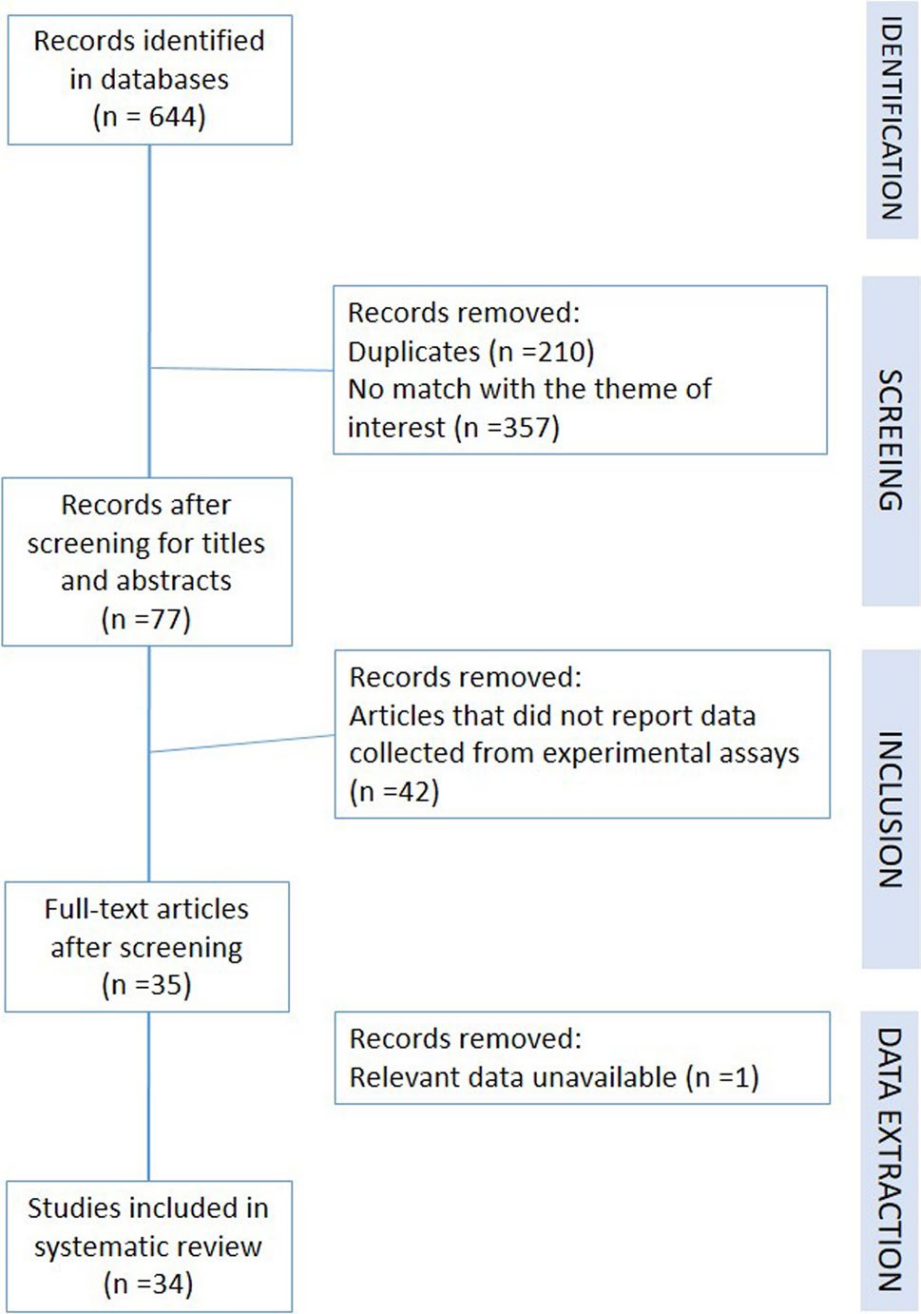
Flow diagram of the literature screening

Based on Peters et al. (2020), we used a four-step strategy to select articles for the review (Fig. 1). We identified relevant articles indexed in the electronic databases based on the search terms “antibiotic” and “phytotoxicity” in combination with Boolean operators. After screening titles and abstracts, we removed duplicate records and articles whose scope did not match the theme of interest. Reviews were also excluded. We included only articles published in English that reported experimental data. After conducting a full-text screening of the articles identified, we retained only those that reported data on the following characteristics: name of the first author, year of publication, type of study, assay characteristics, assay duration, medium, assay conditions, endpoint unit, substance application method, as well as the results and units for all antibiotics and plant species that were assayed (Table 1).

**Table 1:** Data extracted during full-text screening of relevant articles identified for the systematic review

Characteristic	
Type of study	Experimental
Type of assay	Standardised, non-standardised
Medium	Soil, other
Assay conditions	Temperature, light, duration, no. concentrations, no. replicates, no. seeds
Endpoints	Germination, length, growth
Units	mg/kg, mg/L, fresh weight, dry weight
Substance application	Water, solvent, other
Results	EC50, EC10, NOEC
Units	mg/L, mg/kg
Antibiotic tested	Name and class
Plant species assayed	Latin and common name

EC (effect concentration) 50 = concentration at which 50% of effect (e.g. inhibition of germination or growth) is observed compared to the control group; EC 10 = concentration at which 10% of effect is observed compared to the control group; NOEC (no observed effect concentration) = is the highest tested concentration that failed to give a result significantly different from that in the control

Studies were classified into two, non-overlapping groups based on the type of data collected: one group consisted of germination and growth studies that were performed on soil samples and reported results as mg/kg, while the other consisted of germination studies that were performed on other substrates and reported results as mg/L.

**Fig. 2:**
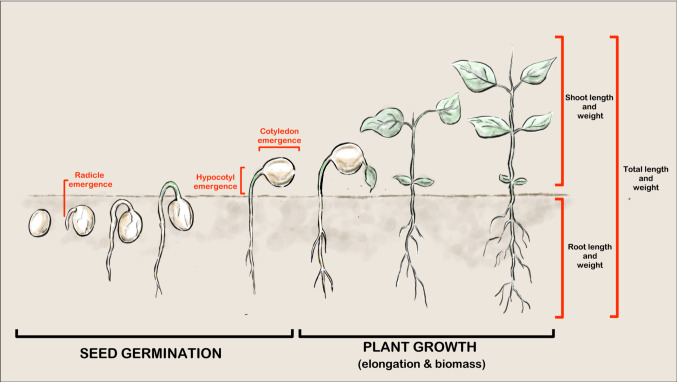
Illustration of the ecotoxicological endpoints of seed germination and plant

Data were included in the two groups using an approach similar to that of Christl et al. (2019). Data were included on seed germination (radicle, hypocotyl or cotyledon emergence), elongation growth (root, shoot and total length), and biomass growth (root, shoot and total weight) (Fig. 2). Biomass data were included without differentiating between wet or dry weight. For endpoint data reported as “greater than” the highest rate or “less than” the lowest rate, “greater than” values were multiplied by 2 and “less than” values were divided by 2, based on the approach of Christl et al. (2019). Based on the same approach, non-observed effect concentrations (NOECs) were converted into effect concentration 10 (EC10) values by assuming that EC10 > NOEC. NOEC values ≥ EC10 were multiplied by 2, and NOEC values < EC10 were divided by 2. Endpoints from multiple tests on the same plant species and antibiotic were merged as their geometric means in order to avoid bias due to more frequent testing of certain species.

After merging all the available data, we initially compared the sensitivity of crop and wild species using the ECx endpoint (EC50, EC10) for each measured variable (seed germination, elongation growth, and biomass growth).

For this purpose, we used the quotient approach proposed by Christl et al. (2019), which uses two reference points (RPs) recommended for risk assessment in the “Guideline on the plant testing strategy for veterinary medicinal products” (EMA/CVMP/ERA/689041/2015): (a) RPgeo, which is reported as the geometric mean and can protect 50% of the species; and (b) RPmin, which is reported as the hazardous concentration for 5% of the population (HC5) or 5^th^ percentile of the species sensitivity distribution (SSD), corresponding to protection of 95% of the species. For those groups or variables with at least three different points on the SSD, namely three EC values from three different plant species-antibiotic combinations, calculations were performed using ETX 2.0 software (Van Vlaardingen et al. 2004). Each resulting RP value for crop species was divided by the corresponding RP value for wild species. In this approach, quotients above 1 indicate that wild species are more sensitive than crop species, while quotients below 1 indicate the opposite.

Although we would have preferred to compare endpoints across different classes of antibiotics and plant families, we were unable to do this for lack of sufficient data: rarely were data on a given antibiotic available for at least three crop and three wild species. Therefore, differences in toxicity among classes of antibiotics and plant families were explored for the merging crop and wild data from studies on plant growth (mg/kg), elongation growth and biomass growth, which are more sensitive parameters than seed germination (Ghava et al. 2015; Pan and Chu 2016; Bellino et al. 2018; Liu et al. 2009a; Wang et al. 2019). A linear mixed effects (LME) model (Lindstrom and Bates 1988) was used in which ECx were considered as random factors in order to control the differences in the incidence values due to intrinsic features of each antibiotic class or plant family. When the effects were significant based on 95% confidence intervals, a post hoc Tukey test (HSD) was performed using the emmeans R package (Lenth et al. 2019).

## Results and Discussion

### Characteristics of Included Studies

A total of 644 studies were identified from the databases examined, including 206 from PubMed, 253 from Scopus, and 185 from Web of Science (Fig. 1). We were unable to identify any additional studies from Google Scholar. After a detailed assessment based on the eligibility criteria and availability of data, 34 unique studies were selected for further analysis.

The characteristics of the studies included in this systematic review are listed in Table 2. Overall, these studies were classified into two groups: those evaluating seed emergence, and those evaluating plant growth. In the first group, seed emergence was evaluated using standardised guidelines developed by international organisations such as the Environmental Protection Agency (EPA), the American Society for Testing and Materials (ASTM) and the International Seed Testing Association (ISTA). In these studies, all assays were performed in a Petri dish with filter paper, and seed growth occurred mainly in the dark in culture media other than soil. Seed emergence and seed growth (radicle, hypocotyledon, and cotyledon length; mg/L) were recorded within 14 days after the start of the assay, mostly in the first week (Fig. 2). In five studies, we found further information on plant growth after 14 days using sand as a culture medium. The second group of studies evaluated plant growth on the basis of the OECD 208 standardised test. Here, all assays were performed in the soil, plant growth occurred mainly under light/dark conditions, and seed emergence and plant growth (length and fresh/dry biomass; mg/kg) were recorded at 28 days after the start of the assay (Fig. 2).

**Table 2:** Characteristics of studies included in the systematic review on the phytotoxic effects of antibiotics on plant species

Reference	Sudy id	Assay id	Method	Duration (d)	Medium	Light	Temp (ºC)	No. Seeds	Substance application	No. and range of concentrations	Replicates	Unit
Timmerer et al. 2020	1	1	Phytobiotest MBT	5	Filter paper	Dark	23	9	Citric acid buffer	5–11	4	mg/L
Pino et al. 2016	2	1	OECD 1984	5	Filter paper	Dark	22	20	Water	5	3	mg/L
Hillis et al. 2011	3	1	ASTM 2003	5–7	Filter paper	Dark	24	10	Water	6	5	mg/L
Tasho et al. 2020	4	1	ISTA 1985	6	Soil	Dark	25	5	Water	9	3	mg/kg
		2	Non- standardised	15	Soil	Light/Dark	25	5	Manure	9	3	mg/kg
Luo et al. 2019a	5	1	Non-standardised	2	Filter paper	Dark	25	20	Water	7	4	mg/L
		2	Non- standardised	2	Filter paper	Dark	25	160	Water	7	0	mg/L
Luo et al. 2019b	6	1	Non- standardised	2–3	Filter paper	Dark	25	160	Water	7	0	mg/L
Rede et al. 2019	7	1	EPA 2012	5	Soil	Light/Dark	24	20	Water	9	9	mg/kg
Litskas et al. 2019	8	1	OECD 1984	21	Soil	Light/Dark	25	20	Water	5	3	mg/kg
Parente et al. 2018	9	1	OECD 1984	16	Soil	Light/Dark	25	5	Water	7	4	mg/kg
Elezz et al. 2019	10	1	Non- standardised	6	Filter paper	Light/Dark		10	Water	5	4	mg/L
Wieczerzak et al. 2018	11	1	Phytobiotest MBT	3	Cotton wool	Dark	23	10	Water	5	3	mg/L
Menezes-Oliveira et al. 2018	12	1	ISO 2012	21	Soil	Light/Dark	20	10	Acetone	6	4	mg/kg
Dipshika and Mehta, 2018	13	1	Non- standardised	7	Filter paper	Light/Dark	25	30	Water	6	3	mg/L
Bellino et al. 2018	14	1	Non- standardised	10	Filter paper	Dark	25	20	Water	5	5	mg/L
		2	Non- standardised	7	Filter paper	Dark	25	20	Water	5	0	mg/L
Litskas et al. 2019	15	1	OECD 1984	21	Soil	Light/Dark	25	5	Water	5	4	mg/kg
Pan and Chu. 2016	16	1	ASTM 2003	5–7	Filter paper	Dark	25	20	Water	6	5	mg/L
Orzoł and Piotrowicz-Cieślak 2017	17	1	Phytobiotest MBT	7	Filter paper	Dark	23	10	Water	8	4	mg/L
		2	Phytobiotest MBT	12	Filter paper	Dark	23	10	Water	8	4	mg/L
Rydzyński et al. 2017	18	1	Non- standardised	30	Soil	Light/Dark	19_23	300	Water	5	1	mg/kg
Riaz et al. 2017	19	1	Non- standardised	2	Filter paper	Dark	26		Water	8	3	mg/L
		2	Non- standardised	20	Sand	Light/Dark	25_21		Water	3	3	mg/L
Minden et al. 2017	20	1	Non-standardised	14	Filter paper	Dark	24	100	Water	3	9	mg/L
		2	Non- standardised	56	Filter paper	Light/Dark	24	10	Water	3	9	mg/L
Richter et al. 2016	21	1	OECD 1984	28	Soil	Light/Dark	22	20	Acetone		4	mg/kg
Eluk et al., 2016	22	1	ASTM 2003	7	Filter paper	Dark	25	10	Water	4	5	mg/L
Ghava et al. 2015	23	1	Non- standardised	12	Filter paper	Dark	24	5	Water	6	3	mg/L
Furtula et al. 2012	24	1	EC 2005	14	Soil	Light/Dark	24	10	Water	8	6	mg/kg
Xie et al., 2011	25	1	Non- standardised	3	Water	Dark	25	600	Water	10	0	mg/L
Wang et al. 2019	26	1	Non- standardised	4	Filter paper	Dark	25	10	Water	5	3	mg/L
Pannu et al. 2012	27	1	Non- standardised	10	Soil	Light/Dark	25	30	Methanol	3	4	mg/kg
Liu et al. 2009a	28	1	ISTA 1985	4–5	Filter paper	Dark	25	15–20	Water	7–8	3	mg/L
		2	OECD 1984	20	Soil	Light/Dark	25	8–10	Water	7–8	3	mg/kg
Liu et al. 2009b	29	1	OECD 1984	20	Soil	Light/Dark	25	1_8	acetone	7		mg/kg
Jin et al. 2009	30	1	Non- standardised	2	Soil	Dark	25	15	Water	7	3	mg/kg
		2	Non- standardised	2–5	Soil	Dark	25	15	Water	7	3	mg/kg
Migliore et al. 2003	31	1	Non- standardised	17–37	agar	Light	20	144_206	Sodium hydroxide	3	?	mg/L
Migliore et al. 1995	32	1	Non- standardised	17–27	agar	Light	20	30_60	Water		10	mg/L
Sidhu et al. 2019	33	1	Non- standardised	15	Soil	Light/Dark	25	2_5	Water	3	3	mg/kg
Hillis et al. 2008	34	1	Non- standardised	14	M medium	Dark	26	6	Methanol	5	6	mg/L
		2	Non- standardised	21	M medium	Dark	26	6	Methanol	5	6	mg/L
		3	Non- standardised	28	M medium	Dark	26	6	Methanol	5	6	mg/L

The first group had a larger number of studies (*n* = 20) and more data on toxicity, but their results did not resemble in vivo conditions. The principle of the seed emergence assay is to determine seed vigour, which can reliably predict field performance. This assay is usually applied to monitor the viability of stored seed collections, but it has also been recommended for obtaining information on the germination requirements of threatened species (Clemente 2017). Seeding is a conservative process, and the seed coat acts as a barrier to protect the plant embryo from the negative impacts of environmental contaminants such as pharmaceuticals (Hillis et al. 2011; Rede et al. 2019).

In contrast, the second group had fewer studies (*n* = 14), but was more representative of field conditions since the studies used soil as the medium. It is widely accepted that elongation and vegetative parameters are sensitive endpoints to evaluate phytotoxic effects caused by the physical interaction of roots with antibiotics and other soil contaminants (Minden et al. 2017).

### Plant Species and Antibiotics

We collected 169 data records on plant species, antibiotics, and toxicity endpoints from studies performed in soil (mg/kg) and 275 data records from studies performed in other media (mg/L). This is one of the limitations of our study, since each of these groups accounted for < 10% of the data on PPPs that were analysed by Christl et al. (2019). Furthermore, most of the data analysed in the present study were from crop species: the data from wild species accounted for < 1% of the data in each group, much less than the 60% in the analysis by Christl et al. (2019). It is clear that the effects of antibiotics on plants, particularly non-crop species, have received very little attention (Minden et al 2017).

Data records were available for 12 antibiotic classes (aminoglycosides, bisphenols, diaminopiridines, fluoroquinolones, ionophoric, lincosamides, macrolides, penicillines, phenicol, quinolones, sulfonamides and tetracyclines) and eight plant families (Apiaceae, Asteraceae, Brassicaceae, Cucurbitaceae, Fabaceae, Liliaceae, Poaceae and Solanaceae) (Table 3). The most frequently assayed antibiotics with endpoint data (ECx) from crop and wild species were tetracyclines (total n / crop n / wild *n* = 99 / 96 / 3), sulphonamides (75 / 73 / 2), quinolones (45 / 44 / 1), macrolides (66 / 60 / 6), and penicillins (29 / 26 / 3). Nearly all these antibiotic classes are top-sellers in the EU, with tetracyclines accounting for 32.6% of antibiotic sales; penicillins, 28.8%; sulphonamides, 9.8%; and macrolides, 7.9% (EMA 2019).

**Table 3:** Antibiotics assayed in the germination studies (mg/L) and growth studies (mg/kg) included in the systematic review on the phytotoxic effects of antibiotics on plant species

		Crop data (mg/L)	Crop data (mg/kg)	Wild data (mg/L)	Wild data (mg/kg)
Class	Antibiotic	Study ID	Study ID	Study ID	Study ID
Aminoglycosides	Kanamycin	22			
	Spectinomycin	14	4		
Bisphenols	Triclosan		27, 29		
Diaminopiridines	Trimethoprim	2, 3, 28	28		
Fluoroquinolones	Enrofloxacin	1, 10, 19, 22, 26, 31	30		
Ionophoric	Monensin	34	12		
	Salinomycin		24		
Lincosamides	Lincomycin	3			
Macrolides	Azithromycin		33		33
	Erythromycin	16			
	Spiramycin	14			
	Tylosin	3, 10, 28, 34	21, 28		21
Penicillines	Amoxicillin	3, 13, 23	15, 7		15
	Penicillin	20		20	
Phenicol	Chloramphenicol	11, 14, 16			
	Florfenicol		21		
Quinolones	Ciprofloxacin		9, 18, 33		33
	Levofloxacin	3, 17, 19, 23, 34			
	Norfloxacin	16			
	Ofloxacin	26			
Sulfonamides	Sulfadiazine	1, 20	30	20	
	Sulfadimethoxine		4		
	Sulfamethazine	3, 16, 28	28		
	Sulfamethoxazole	3, 28	28		
	Sulfamonomethoxine		30		
	Sulphadimethoxine	32			
Tetracyclines	Chlortetracycline	3, 28, 34	28		
	Doxycycline	26, 36	8		
	Oxytetracycline	3, 11, 26, 34	4, 9		9
	Tetracycline	1, 2, 3, 5, 6, 13, 16, 20, 23, 25, 28, 34	18, 28	20	

The most frequently evaluated plant families with data on crop and wild species were Poaceae (total n / crop n / wild *n* = 100 / 193 / 7), Fabaceae (71 / 66 / 5) and Brassicaceae (56 / 53 / 3) (Table 4). There were no data on wild species of any of the other plant families. The most common crop species observed were the two cereals *Oryza sativa* and *Triticum aestivum* (Poaceae), as well as *Brassica campestris* and *Brassica napus* (Brassicaceae), and *Lupinus luteus* and *Phaseolus vulgaris* (Fabaceae). Most of these crops are listed in Annex 2 of the OECD 208 Terrestrial Plant Test, which is the assay recommended in the “Guideline on the plant testing strategy for veterinary medicinal products” (EMA/CVMP/ERA/689041/2015). These species are common crop species in the EU, except *Lupinus luteus*, which is a forage crop whose cultivation has decreased greatly in recent decades. Poaceae cereals, including rice, are the main crops grown in the EU-28 (Eurostat 2017), and they occupy 32.3% of the total arable land. Among these species, rice (*Oryza sativa*) accounts for > 3% of cropland, while wheat (*Triticum aestivum*) accounts for 46%. Pulses (Fabaceae) and vegetables (Brassicaceae) are grown, respectively, on 1.2% and 1.1% of EU cropland.

**Table 4:** Plant species assayed in the germination (mg/L) and growth studies (mg/kg) included in the systematic review on the phytotoxic effects of antibiotics on plant species

Family	Specie	Common Name	Study Id Crop Data (mg/L)	Study Id Crop Data (mg/kg)	Specie	Common Name	Study Id Wild Data (mg/L)	Study Id Wild Data (mg/kg)
Apiaceae	*Daucus carota*	Carrot	3, 16, 34	4				
	*Lactuca sativa*	Lettuce		33, 27, 4, 7, 15				
Asteraceae	*Cichaorium endivia*	Sweet oat	28					
	*Lactuca sativa*	Lettuce	2, 3, 16, 31					
Brassicaceae	*Raphanus sativus*	Radish		27, 9	*Capsella bursa-pastoris*	Shepherd’s purse	20	
	*Brassica campestris*	Cabbage	26	30				
	*Brassica rapa*	Cabbage	5, 6	24, 30, 12				
	*Brassica oleracea*	Cabbage	6					
	*Brassica napus*	Oilseed rape	20, 26	21				
	*Sinapis alba*	Mustard	1	21				
	*Raphanus sativus*	Radish	31	33				
Cucurbitaceae	*Cucurbita pepo*	Pumpkin		8, 15				
	*Cucumis sativus*	Cucumber	16, 26, 28, 31	29, 28				
Fabaceae	*Lupinus luteus*	Yellow lupin	17		*Trifolium pratense*	Red cover		21
	*Medicago sativa*	Alfalfa	3	18				
	*Phaseolus vulgaris*	Common bean	31	21, 8				
	*Pisum sativum*	Pea	32	8, 15				
Liliaceae	*Allium cepa*	Onion		21, 9				
Poaceae	*Avena sativa*	Oat		21	*Apera spicaventi*	Loose silky-bent	20	
	*Hordeum vulgare*	Barley	10		*Festuca arundinacea*	Grass		33, 15
	*Panicum miliaceum*	Proso millet	32		*Lolium perenne*	Ryegrass		9
	*Paspalum notatum*	Bahia grass		27				
	*Oryza sativa*	Rice	13, 28	28, 29				
	*Sorghum bicolor*	Sorghum	11					
	*Triticum aestivum*	Wheat	19, 20, 22, 23, 25	30, 12				
	*Zea mays*	Corn	32	8, 15				
Solanaceae	*Solanum lycopersicum*	Tomato	14, 16	21, 8, 30, 15				
	*Capsicum annum*	Pepper		4				

The wild species analysed were *Apera spicaventi*, *Festuca arundinacea,* and *Lolium perenne* (Poaceae), *Capsella bursa-pastoris* (Brassicaceae), and *Trifolium pratense* (Fabaceae). They are common weed species, or species found in the margins of fields in Europe; in some cases, they are native (or cultivated) forage crops, meadow, grassland, or pasture species (Polunin 1977; Gómez 2008). Floral diversity in crop margins can play a relevant ecological role in the agricultural landscape by providing a niche for invertebrates and serving as an important food source for birds (Vickery et al. 2009). None of the wild species analysed in the present study, except *Trifolium pratense*, is listed in Annex 3 of the OECD 208 Terrestrial Plant Test.

**Fig. 3:**
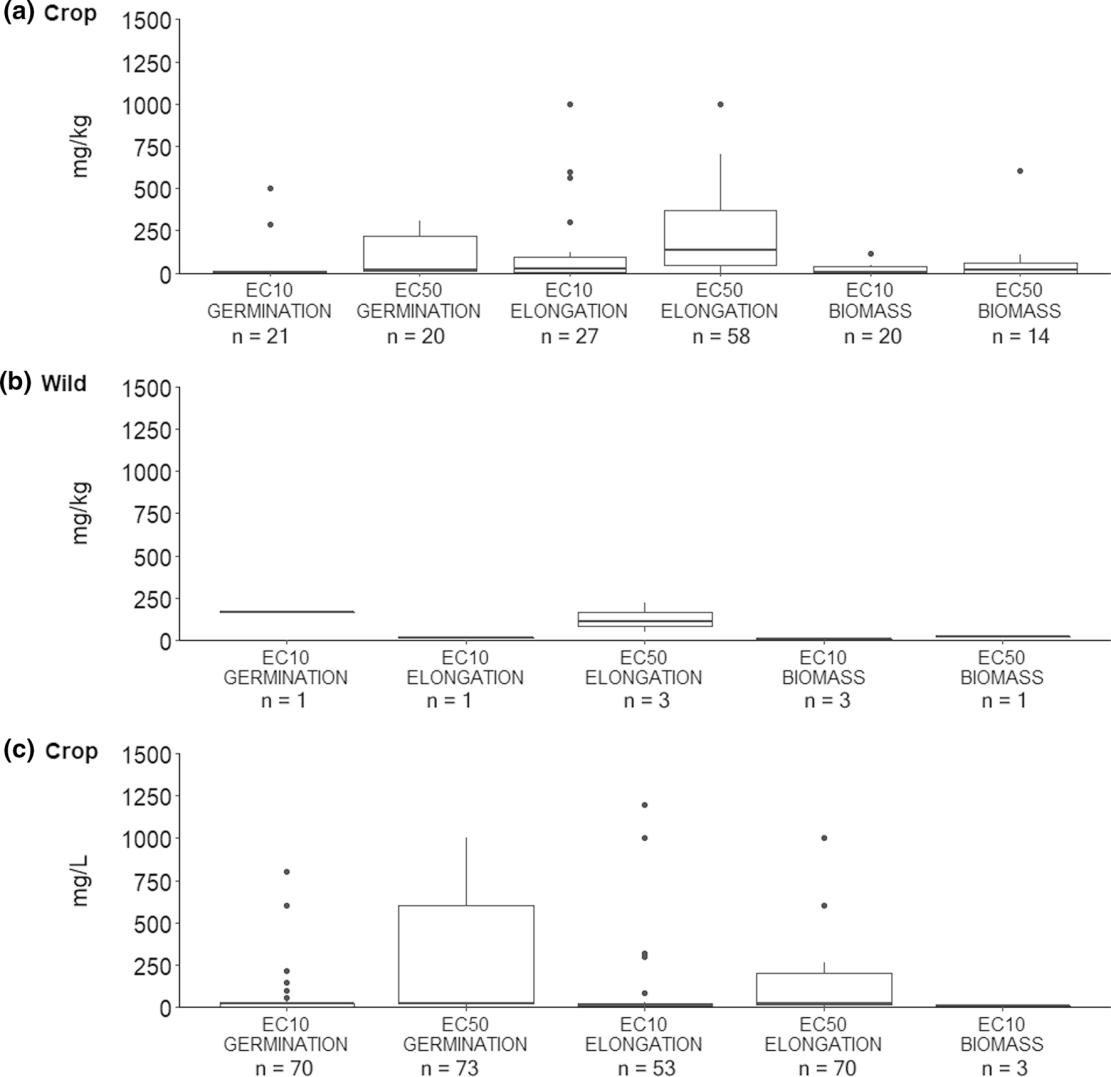
Endpoints (ECx) for each variable for **a** crop species (mg/kg soil), **b** wild species (mg/kg soil) and **c** crop species (mg/L solution)

Native species are expected to show more variation in sensitivity than crop species (Olszyk et al. 2008). Based on the endpoint data (ECx) collected from the plant growth studies performed on soil (mg/kg), wild plant toxicity data fell within the range of crop plant toxicity data (Fig. 3). However, the ranges plotted for crop species were broader than those for wild species, which may reflect the lack of data on wild species.

### Quotient Approach

Based on the quotient approach, we calculated the differences in sensitivity between crop and wild plant species (Table 5). We were unable to calculate average quotients for the data on seed emergence (mg/L), since they did not include at least three different EC50 and three different EC10 values from wild plant species. In contrast, we found differences in sensitivity when we assessed the datasets from the studies on plant growth (mg/kg). When we compared endpoints for elongation, we found that wild species were more sensitive (Q > 1) but when we compared endpoints for biomass, we found that wild species were less sensitive (Q < 1). However, due to the lack of data on wild species (*n* = 3), we could not detect reliable differences in sensitivity between crops and wild species.

**Table 5:** Sensitivity of crop and wild species to antibiotics based on quotients (Q) calculated from reference points (RF) of toxicity data (ECx) on plant growth. Quotients above 1 indicated that wild species were more sensitive than crop species, while quotients below 1 indicated the opposite

Group or variable		Seed germination		Growth (elongation)		Growth (biomass)	
		EC50	EC10	EC50	EC10	EC50	EC10
CROP	n	20	21	58	27	14	120
	RPgeo^(a)^ mg/kg	42.36	3.19	110.39	15.58	8.56	3.08
	RPmin^(b)^ mg/kg	1.21	0.012	4.12	0.213	0.14	0.06
WILD	n	0	1	3	1	1	3
	RPgeo^(a)^ mg/kg	–	–	107.70	–	–	3.30
	RPmin^(b)^ mg/kg	–	–	0.25	–	–	0.25
QUOTIENT	Qgeo^(c)^	–	–	1.6	–	–	0.9
	Qmin^(d)^	–	–	16.36	–	–	0.21

(a) RPgeo, geometric mean and can protect 50% of the species

(b) RPmin, hazardous concentration for 5% of the population (HC5) or 5^th^ percentile of the species sensitivity distribution (SSD), corresponding to protection of 95% of the species

(c) Qgeo, Quotient value based on geometric mean values

(d) Qmin, Quotient value based on 5th percentile of the species sensitivity distribution (SSD) values

### Wild Species Sensitivity

Several studies comparing the effects of antibiotics on crop and wild species from the same plant family have reported that antibiotics may be equally or more harmful to wild plant species than to crop species. One study on Poaceae species reported no significant differences in sensitivity to amoxicillin between crop species (*Zea mays*) and wild species (*Festuca arundinacea*) (Litskas et al. 2019). However, the authors of that study highlighted that amoxicillin can degrade rapidly in soil, decreasing the risk of acute toxicity in plants. Another study reported that tylosin negatively affected emergence and growth of Fabaceae species, and that non-crop species (*Trifolium pratense*) were more sensitive than crop species (*Phaseolus vulgaris*) in terms of EC10 (7.7 vs. 9.1 mg/kg) and EC50 (23.5 vs. 107 mg/kg) (Richter et al. 2016).

A study on plant species from the Poaceae and Brassicaceae families found that exposure to different antibiotics (penicillin, sulfadiazine, and tetracycline) at concentrations similar to those detected in the soil did not adversely affect the germination rate of crop or wild species (Minden et al. 2017). Nevertheless, exposure to those antibiotics did delay germination and affected plant growth at later stages (e.g. canopy and chlorophyll production). These effects were stronger in non-crop species (*Capsella bursa-pastoris;* Brassicaceae and *Apera spicaventi;* Poaceae) than in crop species (*Brassica napus;* Brassicaceae and *Triticum aestivum;* Poaceae). The results of that study indicate that antibiotics can affect the growth of wild plant species to a larger extent than they affect the growth of crop species. This can affect the composition of plant communities at field margins, which may trigger changes in species composition and affect biodiversity in the region (Minden et al. 2017).

The hypothesis that wild species are intrinsically more sensitive to PPPs than crop species has been tested (Christl et al. 2019). After conducting a critical review of available data on wild and crop species and statistically analysing the differences in their intrinsic sensitivity to such products, those authors found no consistent differences between the two groups of plants. In fact, crop species were found to be slightly more sensitive than wild plant species. Our review used a similar approach to analyse the effects of antibiotics on crop and wild species, but it could not arrive at a clear. One major constraint was the lack of published data on wild plant species. ERAs of veterinary medicines can contain additional sources of data, but we were unable to access such data. Moreover, a majority of pharmaceutical veterinary medicine products (> 95%) are considered to have limited environmental release, resulting in low tier (Phase I) risk assessments that do not require the analysis of ecotoxicological data (Fabrega and Carapeto 2020).

### Plant Families and Antibiotic Class Sensitivity

**Fig. 4:**
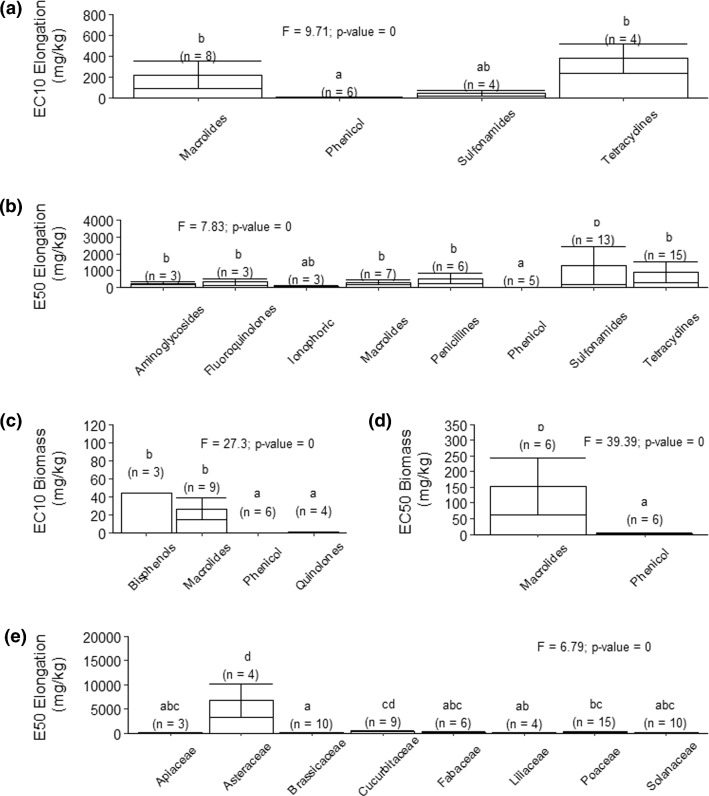
Differences in toxicity among classes of antibiotics: **a** elongation EC10 (mg/kg), **b** elongation EC50 (mg/kg), **c** biomass

Data did show significant differences among classes of antibiotics based on the EC50 and EC10 values of elongation growth and biomass growth (Fig. 4). However, such data often came from different antibiotic classes, and data for a given class were often lacking (*n* < 3). Nevertheless, analysis based on elongation showed that phenicol was the most toxic class of antibiotics, whereas macrolides and tetracyclines were the least toxic. Analysis based on biomass was similar to that based on elongation, except for tetracyclines, due to the lack of data for this antibiotic class. Previous studies have reported variability in antibiotic toxicity to plants. For example, Richter et al. (2016) observed that six plant species (*Allium cepa, Avena sativa, Brassica napus, Synapsis alba and Solanum lycopersicum* and *Phaseolus vulgaris*) differed by approximately two orders of magnitude between florfenicol (phenicol) and tylosin (macrolide). Liu et al (2009a) also found lower toxicity for tylosin (macrolide), chlortetracycline and tetracycline than for trimethoprim (diaminopiridine), sulfamethoxazole and sulfamethazine (sulfonamide) in two plant species, *Oryza sativa* (Poaceae) and *Cucumis sativus* (Cucurbitaceae).

Comparison of plant families did not reveal significant results except for EC50 elongation values, which showed the following trend in sensitivity: Brassicaceae > Liliaceae > Apiaceae, Fabaceae, Solanaceae > Poaceae > Cucurbitaceae > Asteraceae. Similar to our results, Liu et al (2009b) reported *Oryza sativa* (Poaceae) to be more sensitive to triclosan than *Cucumis sativus* (Cucurbitaceae). Conversely, Tasho et al. (2020) showed *Daucus carota* (Apiaceae) to be the most sensitive to sulfadiazine, oxytetracycline and streptomycin, followed by *Lactuca sativa* (Asteraceae) and *Capsicum annuum* (Solanaceae). Litskas et al. (2019) evaluated the effect of doxycycline at concentrations up to 110 mg/kg on five plant species: *Pisum sativum* (Fabaceae), *Cucurbita pepo* (Cucurbitaceae), *Solanum lycopersicum* (Solanaceae), *Phaseolus vulgaris* (Fabaceae) and *Zea mays* (Poaceae). That study reported *Solanum lycopersicum* (Solanaceae) to be the only sensitive plant species. Species differences in sensitivity to antibiotics depend on the antibiotic assayed (Richter et al. 2016; Parente et al., 2019), so more research is needed to explore such differences.

Even though we were unable to arrive at a clear conclusion in this review, the findings can contribute to the current state of knowledge concerning the environmental risk assessment of antibiotics. Further work must be conducted to gain a better understanding of the effects of toxicity on wild plants, antibiotic classes and plant families. This is especially important for preserving biodiversity and enhancing natural capital in the EU, given the requirements of the European Green Deal (European Commission 2019).

## Conclusions

Owing to the lack of data on wild species, we were unable to evaluate the differences in sensitivity to antibiotics between crop and wild plant species. We found that for plant taxonomic groups and antibiotic classes with sufficient data, wild species were more sensitive to antibiotics than crop species in terms of elongation growth, yet less sensitive in terms of biomass growth. Previous studies on potential sensitivity differences between wild and crop species have revealed similar results, so further work is required to explore such differences. Understanding these differences is important for ascertaining whether the current approach of basing ERAs solely on crop plants is sufficient to protect plant biodiversity from antibiotic contamination.

Among the different classes of antibiotics evaluated, phenicol was the most toxic class of antibiotics, whereas macrolides and tetracyclines were the least toxic. However, no significant results were found in the comparison across plant families, except for elongation. We found that Brassicaceae and Liliaceae were the most sensitive families, whereas Asteraceae was the least sensitive. Further studies should verify and extend our findings.

More data on plant sensitivity to antibiotics, including the sensitivity of wild species, may become available when the new Regulation (EU) 2019/6 comes into force in January 2022, which will allow the publication of toxicity data for veterinary medicinal products. Currently those submitting environmental risk assessments for veterinary medicinal products are not required to publish such data (Oelkers 2021), which in fact remain the property of the applicants and cannot be used without corresponding commercial or confidentiality agreements (de la Casa-Resino et al. 2021).

## Data Availability

We are unable to provide access to the datasets analysed in this study since many of them are not publicly available and have been published in journals without open access.
